# Paclitaxel, ifosfamide, and cisplatin (TIP) as salvage and consolidation chemotherapy for advanced germ cell tumor

**DOI:** 10.1007/s00432-014-1760-x

**Published:** 2014-07-26

**Authors:** Masahiro Kurobe, Koji Kawai, Takehiro Oikawa, Daishi Ichioka, Shuya Kandori, Ei-ichirou Takaoka, Takahiro Kojima, Akira Joraku, Takahiro Suetomi, Jun Miyazaki, Hiroyuki Nishiyama

**Affiliations:** 1 Department of Urology, Faculty of Medicine, Institute of Clinical Medicine, University of Tsukuba, 1-1-1 Tennodai, Tsukuba, Ibaraki 305-8575 Japan; 2 Department of Urology, Tsukuba Medical Center Hospital, Tsukuba, Japan

**Keywords:** TIP, Salvage, Chemotherapy, Germ cell tumor

## Abstract

**Purpose:**

The purpose of the study was to assess the efficacy of TIP as salvage chemotherapy for germ cell tumor (GCT) patients with relapsed disease or cisplatin (CDDP)-refractory disease and consolidation chemotherapy for patients who responded unfavorably to first-line chemotherapy.

**Methods:**

Forty-three patients with advanced GCT were treated with TIP. Eleven with relapsed disease and five with CDDP-refractory disease received TIP as salvage chemotherapy. The remaining 27 received TIP as consolidation chemotherapy following initial induction chemotherapy. All patients received prophylactic granulocyte colony-stimulating factor.

**Results:**

In total, 116 cycles of TIP were administered with a median of three cycles (range 1–4 cycles) per patient. Before TIP, 33 patients showed elevated tumor marker and 23 patients (70 %) achieved marker normalization with the chemotherapy. One of six (17 %) patients with refractory disease and 5 of 10 (50 %) patients with relapsed disease achieved durable complete response (CR) after TIP with or without surgery. Eighteen of 27 (67 %) patients receiving TIP as consolidation chemotherapy achieved durable CR. Five additional patients were given further chemotherapy and achieved durable CR. Grade 4 leukocytopenia and thrombocytopenia were observed in 91 and 42 % of patients, respectively; all were managed with routine supportive care. Grade 2 and grade 3 sensory neuropathy was observed in 37 and 2 % of patients, respectively.

**Conclusions:**

The TIP was effective for relapsed patients with favorable risk features and selected CDDP-refractory GCT patients. Results of TIP as consolidation for patients with unfavorable response to the initial chemotherapy were also encouraging. The toxicities were mainly myelosuppression and sensory neuropathy.

## Introduction

About 80 % of patients with advanced germ cell tumor (GCT) can currently be cured with cisplatin-based chemotherapy and surgery, but patients who relapse after initial treatment or patients who did not respond completely to chemotherapy have a poor prognosis. One possible approach to improve outcome is high-dose chemotherapy (HDCT) with autologous stem-cell rescue (Einhorn et al. 2007; Kondagunta et al. 2007). Although large retrospective analysis suggests a benefit from HDCT as the first salvage chemotherapy (Lorch et al. 2011), the treatment is not feasible for all patients. Another approach is risk-adapted management in a salvage setting.

The Memorial Sloan-Kettering Cancer Center (MSKCCC) group proposed the following favorable prognostic factors for achieving a complete response (CR) to cisplatin plus ifosfamide conventional-dose salvage therapy: (1) testicular GCT, (2) prior treatment limited to one program or six or fewer cycles of cisplatin, and (3) progression after either a CR or a partial response (PR) with normal serum tumor markers (McCaffrey et al. 1997). Motzer et al. conducted a prospective study with a combination of paclitaxel, ifosfamide, and cisplatin (TIP) as salvage therapy for relapsed patients having those favorable risk features (Motzer et al. 2000). The results were promising: the response rate was 80 %, and progression-free survival was up to 73 % with a median follow-up of 33 months (Motzer et al. 2000). The results were confirmed in a subsequent MSKCC study treating 46 patients with the same regimen (Kondagunta et al. 2005).

Because TIP is less toxic than HDCT, several investigators studied TIP for patients having unfavorable risk factors including refractory disease or more intense prior chemotherapy (Mardiak et al. 2005; Mead et al. 2005). Although overall results were somewhat inferior compared to the MKSCC series, Park et al. reported TIP achieved durable CR, even in some patients with absolutely (CDDP)-refractory disease (Park et al. 2011). However, at present, data on the efficacy of TIP beyond its original indication are limited.

In 2003, we reported preliminary results on TIP for eight patients with advanced GCT (Kawai et al. 2003). The purpose of the study was to test the feasibility of TIP for Japanese patients in a salvage setting and also to the efficacy of TIP as consolidation chemotherapy for patients who had responded unfavorably to first-line chemotherapy. Although only three patients were treated for the latter indication, all patients having extra-pulmonary visceral metastases achieved durable CR. The treatment was better tolerated when compared with our experience of HDCT (Miyazaki et al. 2000) or that of Japanese multicenter study (Miki et al. 2007) for the same indication. Because the results were promising, we have been used TIP not only as salvage but also as consolidation chemotherapy. We retrospectively analyzed and here report the results of 43 patients treated with TIP.

## Patients and methods

### Patients

Forty-three male patients with advanced GCT were treated with TIP at Tsukuba University Hospital between 2000 and 2012. The median age at the treatment was 31 years (range 20–54 years). Forty-one patients had primary testicular tumors, and two patients were diagnosed with extragonadal GCT originating in the retroperitoneum; 40 patients had non-seminoma, and three patients had seminoma. All patients but one had histologically confirmed GCT with measurable disease and elevated serum tumor markers. The remaining patient had a burned-out tumor in the right testis, retroperitoneal lymph node, and lung metastases. He was diagnosed with non-seminoma based on the tumor marker profile. Pretreatment evaluation included a history and physical examination, chest X-ray, serum tumor markers, and routine blood chemistries. Depending on the sites of metastatic disease, all patients underwent computed tomography of the chest and abdomen and/or pelvis.

Initially, paclitaxel, the key drug of the regimen, has not been approved for GCT by the Japanese Ministry of Health, Labor and Welfare (MHLW). Therefore, the use of paclitaxel for patients with GCTs was reviewed and approved by the Committee of the Tsukuba University Hospital Investigative Fund. Subsequently, TIP was registered as salvage chemotherapy regimen for metastatic germ cell cancer by the Cancer Board of the Tsukuba University Hospital. The written informed consent for TIP was obtained from each patient. In Japan, paclitaxel was approved for refractory or relapsed germ cell cancer by NHLW since 2012.

### Treatment program

The TIP consisted of paclitaxel 175 mg/m^2^ by 24-h infusion on day 1, followed by ifosfamide 1.2 g/m^2^ infusion over 2 h and cisplatin 20 mg/m^2^ given over 2 h on days 2–6. The dosages and schedule for cisplatin and ifosfamide administration were identical to the TIP regimen reported by Motzer et al. (Motzer et al. 2000), but the dose of paclitaxel was fixed at 175 mg/m^2^ in the present study. Mesna 240 mg/m^2^ was administered intravenously before ifosfamide infusions and every 4 h thereafter for a total of three doses per day. All patients received prophylactic premedication with 20 mg dexamethasone 12 and 6 h before paclitaxel and intravenous ranitidine and oral diphenhydramine (each 50 mg) 30 min prior to paclitaxel administrations. Standard anti-emetic and hydration protocols were followed. Patients received prophylactic subcutaneous injection of granulocyte colony-stimulating factor (G-CSF) daily from day 7. If the WBC count exceeded 10,000/µl, G-CSF therapy was discontinued. Courses were repeated every 21 days. The subsequent cycle was withheld until the granulocyte count was >500/µl and the thrombocyte count was >75,000/µl.

For salvage therapy, four cycles of TIP were given to patients with relapsed and CDDP-refractory disease. When used as consolidation chemotherapy, TIP was subsequently started after initial induction chemotherapy. In principle, our induction chemotherapy program was three courses of bleomycin, etoposide, and cisplatin (BEP) (Williams et al. 1987 **)** for good-prognosis patients and four courses of BEP for intermediate- and poor-prognosis patients. For patients having risk factors for bleomycin pulmonary toxicity, etoposide and cisplatin (EP) or etoposide, ifosfamide, and cisplatin (VIP) (Nichols et al. 1998) were used as alternative induction chemotherapy. When response to the initial induction chemotherapy was unfavorable, TIP was started with the subsequent treatment cycle and repeated until tumor marker normalization. If toxicities were acceptable, one more cycle of TIP was administrated after tumor maker normalization. In both settings, if tumor maker normalization was not achieved by four cycles of TIP, the treatment was changed to another chemotherapy or marker-positive surgery.

### Evaluation of response and toxicities

Clinical response was evaluated according to the criteria in the General Rules for Clinical and Pathological Studies on Testicular Tumors of the Japanese Urological Association. CR was defined as the disappearance of all evidence of disease when documented by imaging and all tumor marker levels. PR with tumor marker-negative findings (PRm−) was defined as a ≥50 % reduction in the product of perpendicular diameters for each indicator lesion and normalization of previously elevated tumor marker levels. PR with tumor marker-positive findings (PRm+) was defined as a ≥50 % reduction in the product of perpendicular diameters for each indicator lesion, but without complete normalization of tumor marker levels. Patients showing PR to chemotherapy and complete surgical resection of fibrosis, necrosis, and mature teratoma were considered to have complete pathological remission. Progressive disease (PD) was defined as a 25 % increase in the product of perpendicular diameters for any lesion or the appearance of any new lesions. No change (NC) was defined as disease that did not meet any of the above criteria, irrespective of tumor marker normalization.

Survival duration was measured from the date of the initiation of TIP to the last follow-up appointment or until death. Evaluation of toxicities was classified according to the National Cancer Institute Common Toxicity Criteria for Adverse Events, version 4.0 (CTCAE v4.0).

### Statistical analysis

Survival curves were constructed by the Kaplan–Meier method and compared using the log-rank test. In both analyses, the level of significance was set at *P* < 0.05. Statistical analysis was performed using Jmp^®^10 software (SAS Institute Inc., Cary, NC).

## Results

### Patient characteristics

The patients’ backgrounds are summarized in Table 1. Sixteen patients received TIP as salvage chemotherapy. Of them, ten patients were relapsed cases who suffered disease progression after CR to prior chemotherapy and/or surgery. Six patients were considered to have CDDP-refractory disease because tumor progression occurred during prior CDDP-based chemotherapy or within four weeks of the last CDDP-based chemotherapy. As salvage chemotherapy, 11 of 16 patients received TIP as second-line chemotherapy, whereas the other five patients received the treatment as third-line or later chemotherapy.

**Table 1:** Patient characteristics

	No.	%
Median of age (range)	31 (20–54)	
*Primary site*		
Testis	41	95
Retroperitoneal	2	5
*Histology*		
Seminoma	3	7
Non-seminoma	39	91
Not known	1	2
*Sites of disease*		
Lymph node	36	84
Lung	32	74
Liver	15	35
Bone	3	7
Brain	3	7
Others	5	12
*Indication of TIP*		
Elapsed	10	23
Refractory	6	14
Consolidation	27	63
*IGCCCG of consolidation*		
Poor	20	74
Intermediate	4	15
Good	3	11
*No. of prior regimens for relapsed/refractory cases*	16	
Third line or more	2	13
Second line	3	19
Induction	11	69

The remaining 27 patients received TIP as consolidation chemotherapy. At the start of the initial chemotherapy, three patients (11 %) were classified as having a good prognosis, four patients (15 %) as intermediate prognosis, and 20 patients (74 %) as poor prognosis according to the classification defined by the International Germ Cell Cancer Collaborative Group (IGCCCG) (International Prognostic Factor Study Group 1997). The initial chemotherapy was BEP for 20 patients, VIP for two patients, VIP/BEP for two patients, and EP/BEP for three patients. In principle, TIP was introduced when responses to the initial induction chemotherapy were unsatisfactory. Twenty patients (74 %) were marker-positive at the start of TIP, 18 were hCG-positive, and 8 were AFP-positive. The remaining seven patients with normalized tumor markers had unrespectable residual tumors at the start of TIP. TIP was started after four courses of initial chemotherapy for 10 patients and after three courses for 17 patients. In the latter cases, the up-front TIP introduction was selected because marker decline during the three initial courses lead us to expect that one additional course of initial chemotherapy would fail to achieve marker normalization.

### Treatment and toxicity

In total, 116 cycles of TIP were administered with a median of three cycles per patient (range 1–4 cycles). The median number of days between cycles was 21 days (range 21–65 days). The reasons for delays were leukocytopenia in 10 cycles, thrombocytopenia in 15 cycles, allergic reaction to transfusion in two cycles, or radiological examinations or others in seven cycles.

The toxicity profile is shown in Table 2. The toxicity of TIP was considered tolerable except in one patient. This patient developed grade 3 sensory neuropathy at the first course of TIP and refused further treatment. Otherwise, 16 patients developed grade 1 or grade 2 sensory neuropathy. Myelosuppression was the major toxicity. Most patients developed grade 3 or grade 4 leukocytopenia despite routine prophylactic use of G-CSF. Among them, 23 (53 %) patients developed neutropenic fever, but all of whom were successfully treated with empirical broad-spectrum antibiotics. Red blood cell and platelet transfusion were needed for 29 patients (67 %) and 21 patients (49 %), respectively.

**Table 2:** Toxicity (CTCAE v4.0)

	All grade	Grade 3	Grade 4
	*n* (%)	*n* (%)	*n* (%)
*Hematological*			
Leukocytopenia	43 (100)	3 (7)	39 (91)
Thrombocytopenia	42 (98)	14 (33)	18 (42)
Anemia	43 (100)	30 (70)	4 (9)
Febrile neutropenia	23 (53)	23 (53)	0
*Non-hematological*			
Nausea or vomiting	23 (53)	4(9)	0
Neuropathy (sensory)	17 (40)	1 (2)	0
Myalgia/arthralgia	11 (26)	0	0
Acoustic nerve disorder	2 (5)	0	0
AST/ALT	4 (9)	0	0
Dysgeusia	1 (2)	0	0

### Response and survival

Response was assessed in all patients as shown in Table 3. Before TIP, 33 patients showed elevated tumor marker, of them 23 patients (70 %) achieved marker normalization with the chemotherapy. In total, 24 patients (56 %) achieved CR: 6 patients with CR after chemotherapy alone and 18 patients with adjunctive surgery for residual tumor after chemotherapy. In the latter cases, pathological examination revealed necrosis or teratoma in 15 patients and viable germ cell cancer in three patients. The CR rate of patients receiving TIP as consolidation chemotherapy was 67 %, which was higher than that of refractory cases (17 %) or relapsed cases (50 %). However, it is notable that the high CR rate was the effect of combined induction chemotherapy and TIP. There were eight patients (19 %) with PR with tumor marker-negative findings (PRm−) and four patients (9 %) with PR with tumor marker-positive findings (PRm+). The remaining five patients (12 %) and two patients (5 %) exhibited NC and PD, respectively. Seventeen of 19 non-CR patients received further chemotherapy after TIP.

**Table 3:** TIP therapy results

	*n* (%)	*n* (%)	*n* (%)
	Refractory	Relapse	Consolidation
Total	6 (100)	10 (100)	27 (100)
CR to chemotherapy ± resection of necrosis/teratoma	1 (17)	4 (40)	16 (59)
CR to chemotherapy + resection of viable germ cell tumor	0	1 (10)	2 (7)
PRm−	2 (33)	1 (10)	5 (19)
PR 171+	1 (17)	2 (20)	1 (4)
NC	2 (33)	2 (20)	1 (4)
PD	0	0	2 (7)

*CR* complete response, *PRm−* partial response with normalized markers, *PRm+* partial response without normalized markers, *NC* no change, *PD* progressive disease

**Fig. 1:**
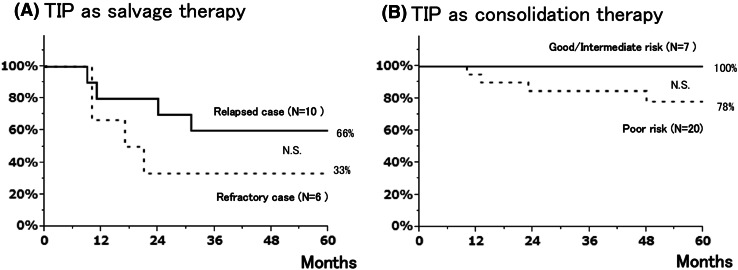
**a** Overall survival rate of patients receiving TIP as salvage chemotherapy for relapsed disease or CDDP-refractory disease, and **b** overall survival rate of patients receiving TIP as consolidation chemotherapy after initial induction chemotherapy according to IGCCCG

As a result, 31 of the 43 patients achieved durable disease-free status. All 31 patients are currently alive without evidence of disease at a median follow-up duration of 58 months (range 19–166 months). As shown in Fig. 1a, the 5-year overall survival (OS) rates of refractory and relapsed cases were 33 and 66 %, respectively. Figure 1b represented the OS of patients who received TIP as consolidation chemotherapy according to the IGCCCG classification. The OS rates of patients with good- or intermediate-prognosis disease were 100 %. The 5-year OS rate of patients with poor-prognosis disease was 78 %.

## Discussion

The TIP was originally developed as first-line salvage chemotherapy for testicular germ cell cancer patients who relapsed after good response (CR or PRm−) to prior chemotherapy (Motzer et al. 2000; Kondagunta et al. 2005). TIP has become one of the most widely accepted conventional-dose salvage chemotherapy in Japan. Recently, Japanese GCT guideline recommended TIP as salvage chemotherapy along with vinblastine, ifosfamide, and cisplatin (VeIP)/VIP. In the present study, we evaluated the activity of TIP for GCT patients in three situations: salvage setting for relapsed cases, salvage setting for CDDP-refractory cases, and the consolidation setting as consolidation chemotherapy. The conditions of the latter two categories are different from original indication for TIP.

In this series, 5 of 10 patients (50 %) with relapsed disease achieved a durable CR after TIP. One additional patient was salvaged with a combination of gemcitabine and oxaliplatin (GEMOX) (Kollmannsberger et al. 2004) after TIP. Consequently, the 5-year OS was 66 %. The durable CR rate was lower than TIP series from MKSCC, but our series includes two patients treated in either a third- or fourth-line setting. One additional patient underwent biopsy of non-responding lymph node metastasis after one cycle of TIP. The pathological diagnosis was primitive neuroectodermal tumor (PNET). Those three patients died of germ cell cancer or PNET. When limited to the remaining seven patients having favorable risk features as defined by MKSCC, the durable CR rate after TIP was 71 %, which was identical to the results of the MKSCC series (Motzer et al. 2000; Kondagunta et al. 2005).

In contrast to relapsed cases, there has been limited information on the efficacy of TIP against CDDP-refractory disease. We treated six patients in this category. It is notable that those patients had other multiple unfavorable risk features including either third- or fourth-line setting (three patients) or progression during prior chemotherapy (four patients). Despite this, two of six patients (33 %) responded to TIP; one patient had CR after TIP and surgery and another patient had PRm- after one cycle of TIP. Despite the favorable initial response in the latter case, we were forced to change TIP to GEMOX due to the development of grade 2 hearing impairment. Because both patients achieved long-term CR, the 5-year OS of patients with CDDP-refractory disease was 33 %. Although patient number of the present study is limited, recently, Park et al. reported the similar efficacy of TIP against relapsed or refractory patients having unfavorable risk features (Park et al. 2011). They reported that 3 of 7 patients (43 %) with CDDP-refractory disease achieved durable CR. These findings might suggest the applicability of TIP for some patients with CDDP-refractory disease. At present, little is known about which subset of patients with CDDP-refractory disease can derive a benefit from TIP. From this point of view, it is notable that two responding patients in our series had responded well (both PRm−) to the last chemotherapy before introduction of TIP, but the results should be interpreted with caution and further studies are needed.

In addition to salvage setting, we tested the efficacy of TIP as consolidation for patients who had shown an unfavorable response to the initial induction chemotherapy. Of them, all seven patients with good- or intermediate-prognosis disease and 11 of 20 (55 %) of patients with poor-prognosis disease achieved durable CR after TIP with or without surgery. Eight of nine non-CR patients were further treated with third-line or more chemotherapy and/or surgery, five patients ultimately achieved durable CR, and three patients died of cancer. The remaining patient with PRm− status underwent RPLND after TIP, but the resection was incomplete. The patient died of progressive teratoma with malignant transformation. Consequently, the 5-year OS rate of patients with poor-prognosis disease was 78 %. It must be emphasized that the high OS rate results from initial chemotherapy, TIP or other chemotherapy regimens in some cases, but the favorable outcomes presented here suggest the benefit of TIP as consolidation chemotherapy. Although a different strategy, several investigators recently tested the efficacy of including paclitaxel in induction chemotherapy. A randomized study comparing four courses of BEP and paclitaxel-BEP (T-BEP) for intermediate risk germ cell cancer showed 12 % superior 3-year progression-free survival with T-BEP, when limited to eligible patients (De Wit et al. 2012). The prospective study of TIP in the first-line setting for patients with intermediate- or poor-risk disease is now ongoing at MKSCC (Voss and Feldman 2011).

Finally, in the present study, we reviewed the medical records with special attention to toxicities during 116 cycles of TIP for 43 patients. To our knowledge, this is the largest report on a toxicity profile of TIP in Japanese patients. Toxicities consisted mainly of myelosuppression, which is in line with previous studies. Most of the patients developed grade 3 or grade 4 leukocytopenia; subsequently, 23 patients (53 %) suffered from febrile neutropenia, but all of them were manageable with empirical broad-spectrum antibiotics. Kondagunta et al. reported that 48 % of 46 patients developed neutropenic fever during TIP using a higher paclitaxel dose of 250 mg/m^2^ (Kondagunta et al. 2005). In the present study, five patients with relapsed or refractory disease received TIP as third-line or later chemotherapy. In consolidation chemotherapy, TIP was subsequently started after initial induction chemotherapy. The situations were different from MKSCC series, where all patients received TIP as second-line chemotherapy after continuous CR to prior CDDP-based regimens. To avoid serious complication, we fixed the dose of paclitaxel at 175 mg/m^2^; nevertheless, a half of patients experienced neutropenic fever. This might be due to the intensiveness of pretreatment in our patients, but there is a possibility that myelosuppression is more severe in Japanese patients. Besides myelosuppression, sensory neuropathy was frequently seen, and one patient experienced grade 3 neurotoxicity. Kondagunta et al. also reported that 4 % of patients developed grade 3 neurotoxicity. In our series, no other non-hematological toxicities over grade 3 were observed. These observations suggest that TIP with paclitaxel at a higher dose is possible in Japanese patients. Because dose-finding study for paclitaxel in TIP regimen was not performed, we are panning the study using 210 mg/m^2^ of paclitaxel, which is the highest dose approved in Japan.

In conclusion, TIP was effective salvage chemotherapy for relapsed patients with favorable risk features and selected CDDP-refractory patients. Results of TIP as consolidation for patients with an unfavorable response to the initial chemotherapy are also encouraging. Toxicities of TIP in these situations were manageable in most of the patients.
